# Electrophysiological Correlates of Blast-Wave Induced Cerebellar Injury

**DOI:** 10.1038/s41598-018-31728-4

**Published:** 2018-09-11

**Authors:** Gokhan Ordek, Ahmet S. Asan, Esma Cetinkaya, Maciej Skotak, Venkata R. Kakulavarapu, Namas Chandra, Mesut Sahin

**Affiliations:** 1 Department of Biomedical Engineering, New Jersey Institute of Technology, Newark, 07102-1982 NJ USA; 2 Center for Injury Biomechanics, Materials, and Medicine (CIBM3), New Jersey Institute of Technology, Newark, 07103-3540 NJ USA

## Abstract

Understanding the mechanisms underlying traumatic neural injury and the sequelae of events in the acute phase is important for deciding on the best window of therapeutic intervention. We hypothesized that evoked potentials (EP) recorded from the cerebellar cortex can detect mild levels of neural trauma and provide a qualitative assessment tool for progression of cerebellar injury in time. The cerebellar local field potentials evoked by a mechanical tap on the hand and collected with chronically implanted micro-ECoG arrays on the rat cerebellar cortex demonstrated substantial changes both in amplitude and timing as a result of blast-wave induced injury. The results revealed that the largest EP changes occurred within the first day of injury, and partial recoveries were observed from day-1 to day-3, followed by a period of gradual improvements (day-7 to day-14). The mossy fiber (MF) and climbing fiber (CF) mediated components of the EPs were affected differentially. The behavioral tests (ladder rung walking) and immunohistological analysis (calbindin and caspase-3) did not reveal any detectable changes at these blast pressures that are typically considered as mild (100–130 kPa). The results demonstrate the sensitivity of the electrophysiological method and its use as a tool to monitor the progression of cerebellar injuries in longitudinal animal studies.

## Introduction

The devastating consequences of severe head injuries are well known to the public. It is also known that undetected mild TBI can be a high risk factor for subsequent injuries and repeated mTBI, whether identified or not, leads to much more serious injuries^1^. Time of intervention is a critical parameter to achieve the best results in the treatment of TBI patients. This requires the knowledge of the time course of injury and its severity. To this end, animal models have been developed to generate better controllable results such as injury severity, type, and location, as well as the age, gender and genetic composition of the subjects, for investigations of immunohistochemical and biomechanical aspects of TBI. Although animal models continue to provide valuable insights into the mechanisms of brain injury, the need to terminate the animals for histological evaluation introduces a significant source of variability by preventing data collection at multiple time points in the same animals during injury progression. Thus, post-mortem techniques rely on statistics to account for inter-animal variations. Cascaded sequelae of the initial and delayed phases of neural injury make it further difficult to determine the temporal course of the injury. Secondary (or delayed) injury mechanisms can last minutes to months including cascaded metabolic, cellular and molecular events that lead to brain cell and tissue damage^2–4^. On the other hand, the electrophysiological technique can provide a powerful tool for multi-point measurements or continuous monitoring of the biomarkers correlated with injury while cascaded changes are taking place in live animals. We developed a novel approach to monitoring various phases of injury using multi-electrode arrays (MEAs) implanted on the cerebellar surface (micro-ECoG technique) to detect any subtle changes in the cortical network excitability. Implanting the rats with MEAs before the blast exposure provided a baseline within the same subject for comparison.

Traditionally, the cerebellum has been considered as a brain center for sensorimotor integration and motor coordination. In recent years the cerebellum has been implicated in cognitive functions and emotions^5,6^. Despite numerous studies, there is still no consensus either on the nature of the information provided by the cerebellar outputs to other brain centers, or how the disruption of these outputs leads to the observed functional deficits after head trauma. The neurologic disabilities that result from a direct insult to the cerebellum include ataxias, tremors, loss of balance and motor skills, and cognitive deficits^7–10^.

To date, the cerebellum has been understudied in the field of TBI research because of the notion that majority of cerebellar deficits occur only by direct impacts on the cerebellum, which rarely occurs in accidents. However, a recent report showed functional and structural cerebellar deficits as a result of blast induced repeated mTBI where the entire brain was affected^11^. Another report suggested a lower threshold for cerebellar injuries in veterans exposed to repetitive blasts^12^. Although diffuse axonal injury (DAI) is the main focus of mTBI research, recent evidence also indicated vulnerability of the synaptic mechanisms to blast injuries^13–16^. Other findings in cerebellar injuries included Purkinje cell (PC) deterioration^17–19^, synaptic disruptions^11,20^ and behavioral deficits^8,17,18^.

Scientific evidence is building up to suggest that mild head injuries, including concussions, can leave permanent damage in the brain especially if they reoccur before the person completely recovers from the first injury^21^. These mild injuries are difficult to study in experimental animals because the damage may not cause the brain cells to show any anatomical changes or complete degenerations, but rather slowing down of their communication with other cells. Furthermore, mild injuries cannot be detected using behavioral measures since the impairments may be too subtle to affect the motor function or cognition or need a prohibitively large sample size to be detected. Here, we propose a highly sensitive electrophysiological method as a tool to monitor the state of on-going cerebellar injury with repeated or continuous recordings of evoked potentials in anesthetized animals.

## Results

**Figure 1:**
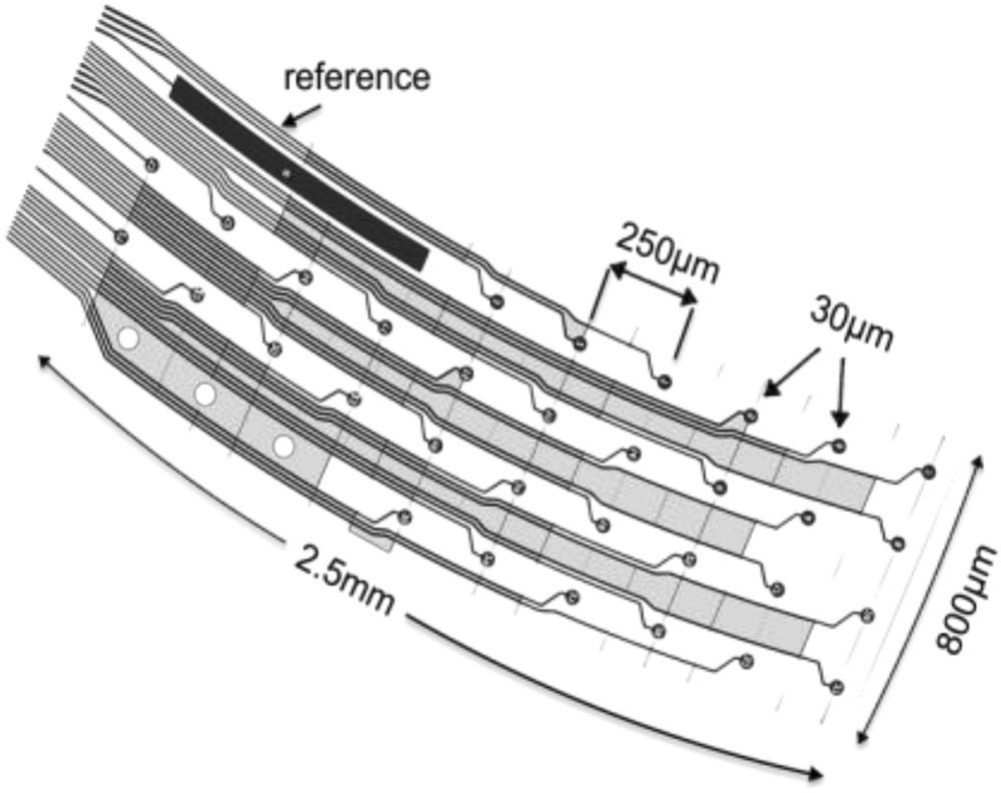
Custom-design multi-electrode array contained 32 Pt contacts (30 µm diam.) that were arranged in 4 rows and 10 columns that cover most of the right PML. The first four contacts are missing in the design on the first row, where the reference contact is, and also on the fourth row.

**Figure 2:**
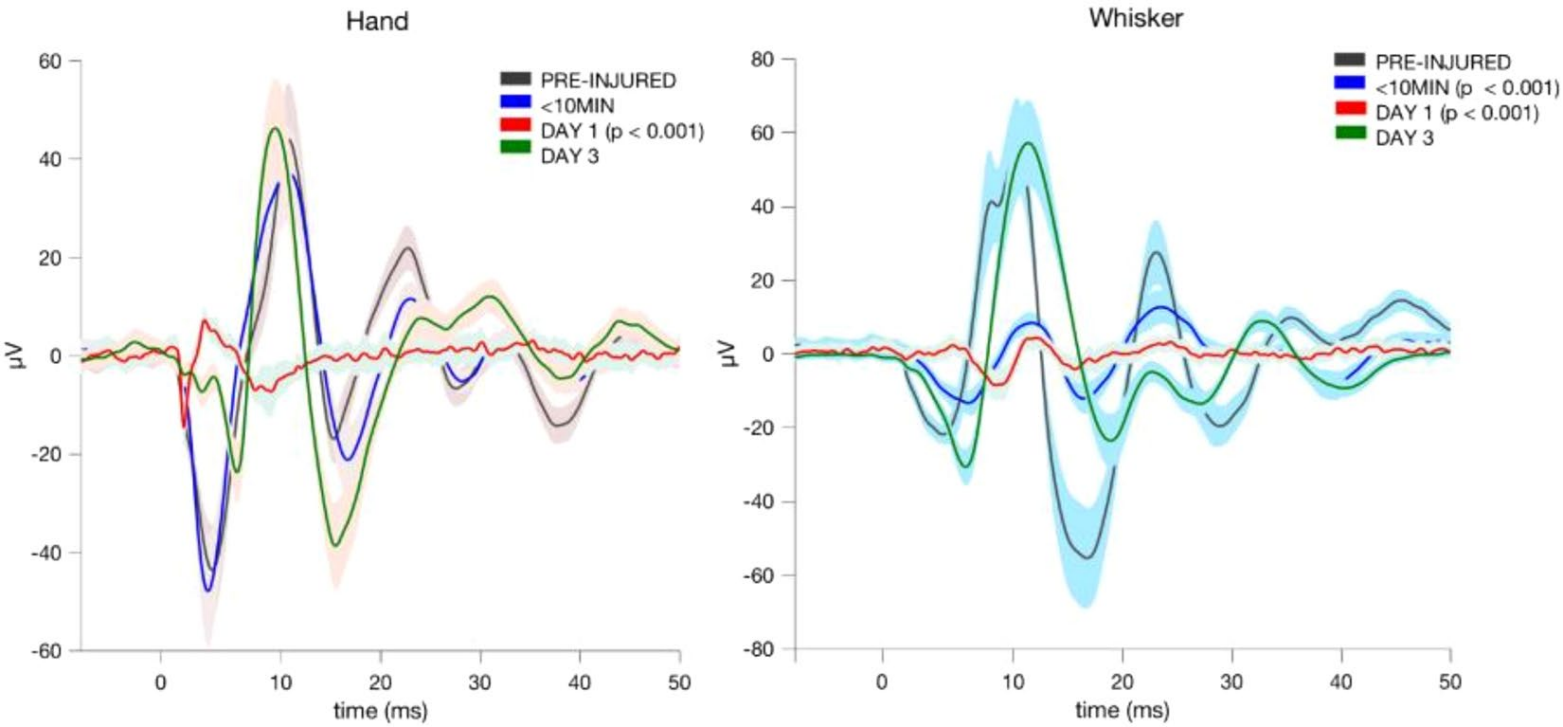
Sample evoked potential (EP) waveforms for hand and whisker stimulations before and after injury in a rat. Stimulus-trigger averaged (STA) signals were filtered at 10–500 Hz, and then averaged across channels. Multiple recordings (4–5 STAs) were averaged for each time point (i.e. each trace) and plotted as mean (solid) ± s.d (shades) from the time of stimulus arrival (t = 0 ms). While pronounced changes in EPs to whisker stimulations occurred immediately after injury (<10 min, T = 9 recordings, p < 0.001), hand EPs indicated significant changes only on the next day (T = 10, p < 0.001).

We first studied the effects of blast injury on the cerebellar local field potentials evoked by dorsal hand and whisker mechanical stimulation using a custom-design multi-electrode array (Fig. 1). Changes in the EP waveforms were shown on the post-injury days compared to the control day for a sample rat (Fig. 2). In general, the cerebellar EPs demonstrated characteristic waveforms that are reproducible. The amplitudes of evoked potentials (EPAs) were in the 120 ± 10 µV-160 ± 20 µV range for the largest deflections (latency of 8–10 ms) as a response to whisker stimulation in the pre-injury period. The largest deflection amplitude drastically dropped down to 10 ± 5 µV immediately after the blast injury (<10 min). The EPs evoked by hand stimulation persisted around the same amplitudes immediately after injury (paired t-test, n = 4 trials, p > 0.5) but decreased substantially on the next day (day-1). The EPs for both hand and whisker stimulations were very small and barely above the baseline noise on day-1 (red traces, 5–8 ± 2–4 µV). On day-3 of injury, both signals showed larger and multiple volleys compared to day-1, which suggested a partial recovery from injury (green trace, 60–80 ± 8–13 µV).

**Figure 3:**
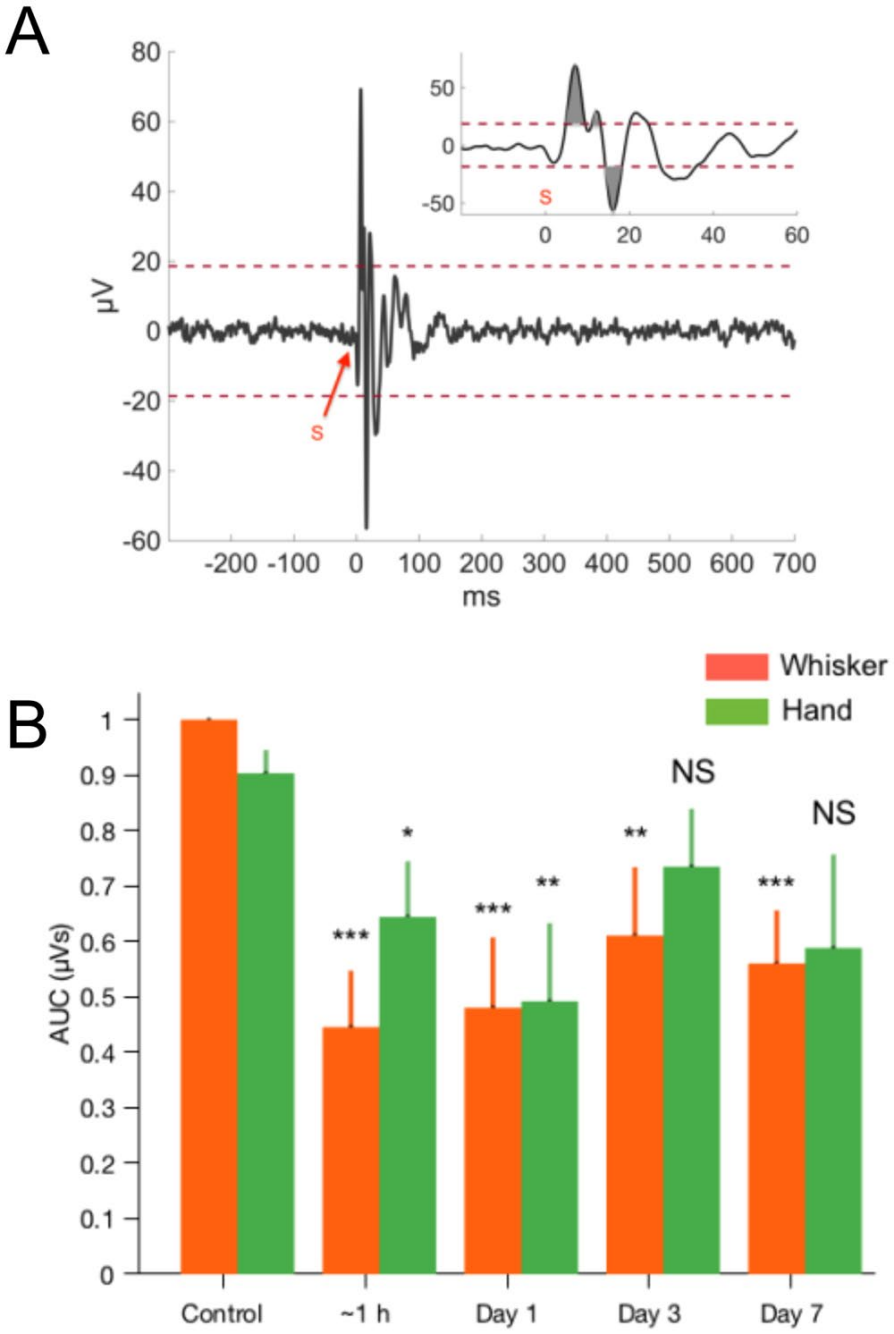
EP quantification by the area under the curve (AUC) method. (**A**) An example of AUC calculation is shown on a stimulus-trigger averaged signal that had a sufficient amplitude to be included in the analysis (threshold ± 20 µV). (**B**) Mean EP changes during the 7 days following blast injury (N = 7 rats). AUCs showed significant drops for both whisker (F(4, 30) = 5.69, p = 0.0016; rmANOVA) and hand evoked potentials (F(4, 30) = 2, p = 0.011; rmANOVA). Bars indicate ± s.d. Significance vs. control (pre-injury) is indicated by the asterisks according to Dunnett’s test. ***P < 0.001, **P < 0.01, *P < 0.05. NS: not significant.

The changes in EPAs were quantified by calculating the area under the curve (AUC) as a measure, which reflects both the amplitude and duration information of a deflection in the EPs. An illustrative example from a control (pre-injury) animal was shown for a single channel of the MEA (Fig. 3A) and in multiple animals for hand and whisker stimulations (Fig. 3B, N = 7 rats). The raw AUC values were normalized by the maximum AUC that was observed in the pre-injury period within each animal individually and then averaged across all animals. The AUC values decreased significantly after the blast exposure during the 7 days after injury (N = 7 rats). Most drastic changes occurred within one hour after injury for the whisker EPs and on the next day for the hand EPs (a decline by ~56% in whisker AUCs at 1 h, p < 0.001 and by 43% in hand AUCs on day-1, p < 0.01; Dunnett’s test). On day-3, AUCs increased slightly compared to day-1 measurements (Whisker ~13%, p < 0.44; Hand ~%24, p < 0.16) similar to the trend in EP amplitude measures in Fig. 2, which suggested a partial recovery. On day-7, the average AUCs were still lower than the control values by ~44% (p = 0.003, Dunnett’s test) and ~32% (p = 0.07, Dunnett’s test) for whisker and hand stimulation respectively. However, the AUC measures that were obtained from day-3 (73.1 ± 10%) and day-7 (58.3 ± 17.1%) for the hand EPs were not significantly different from pre-injury values (90.3 ± 4.3%).

**Figure 4:**
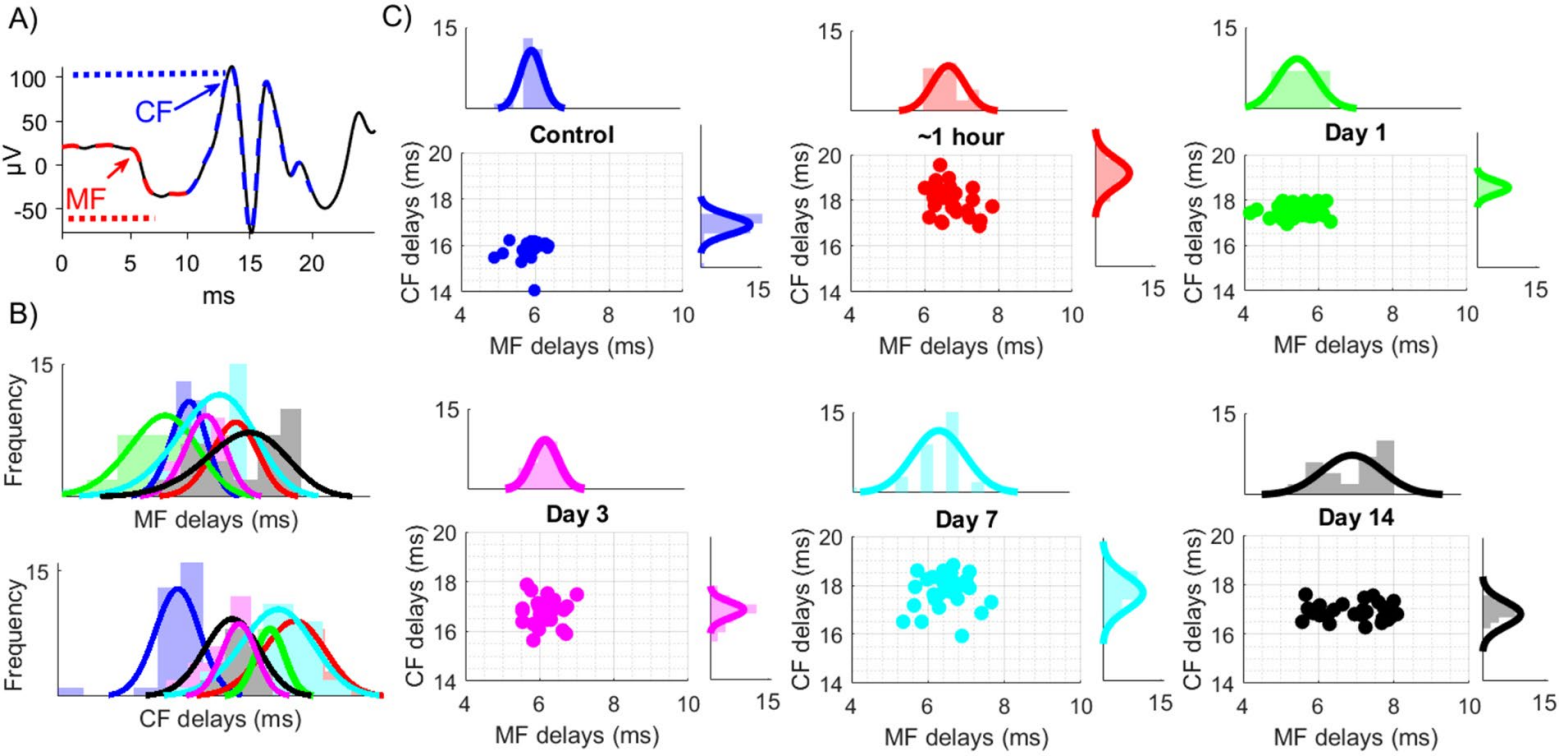
Arrival times of MF and CF mediated local field potentials evoked by mechanical hand stimulation before and after injury. (**A**) Identification of the MF onset and the CF peak, based on arrival times (arrows). Stimulus is at 0 ms. (**B**) MF and CF delays for each array contact were averaged across multiple trials (2–3 trials per day) separately in each day and animal (3 rats indicated by different symbols) and plotted against each other as an x-y chart to show the variation across 31-channels of the recording array. Histograms show day-by-day (color coded) changes in the mean and distribution of the delays. All statistical significances were performed with paired comparisons between pre-injury (control) vs. each day of post-injury. N = 3, Mann-Whitney; *P < 0.01, **P < 0.001, ***P < 0.0001. (**C**) All histograms from different days are merged into a single plot to demonstrate the trends in MF and CF delays day-by-day.

Next, the EPs were segregated into MF and CF mediated potentials as identified by their characteristic latencies from the time of stimulus (Fig. 4A), i.e. 2–10 ms for MF (red) and 12–20 ms for CF (blue). Because hand EPs were more reproducible than that of whiskers, we used only the hand evoked signals in the rest of the analysis. For precise measurements of arrival times, the EPs were spike-trigger averaged across multiple trials in each day for individual channels of the recording array separately. Consequently, each dot in the x-y plots of Fig. 4B represents a single contact out of 31 contacts of the array for the specified day of recording (color coded) in one of the animals. The histograms on the top and the right side of each plot show distributions of the MF and CF arrival times (latencies) across the array contacts in all animals as group data. The results suggest that there was a transient lengthening of MF onset latencies from the control values of 5.5–6.5 ms to 6–8 ms within the first hour of injury, which was followed by a decrease to the 4–6 ms range on day-1. On the following days, there was a progressive increase in MF delays with mean values of 6.05 ms, 6.6 ms, and 7.05 ms on days 3, 7, and 14 respectively. The variance of the MF latencies across the channels was much wider on the post-injury days compared to the pre-injury values. Conversely, the CF peak delays started increasing immediately after injury (1 h) from a range of 15–16 ms and reached to 16–20 ms with a mean of ~19 ms, and stayed higher for the rest of the study period without a specific pattern in the trend. Most notably, significant changes were observed both in MF and CF arrival times within the first hour, although they moved in different directions specifically on day-1. Overall, these results suggest that the injury induces differential effects on the MF and CF arrival times.

**Figure 5:**
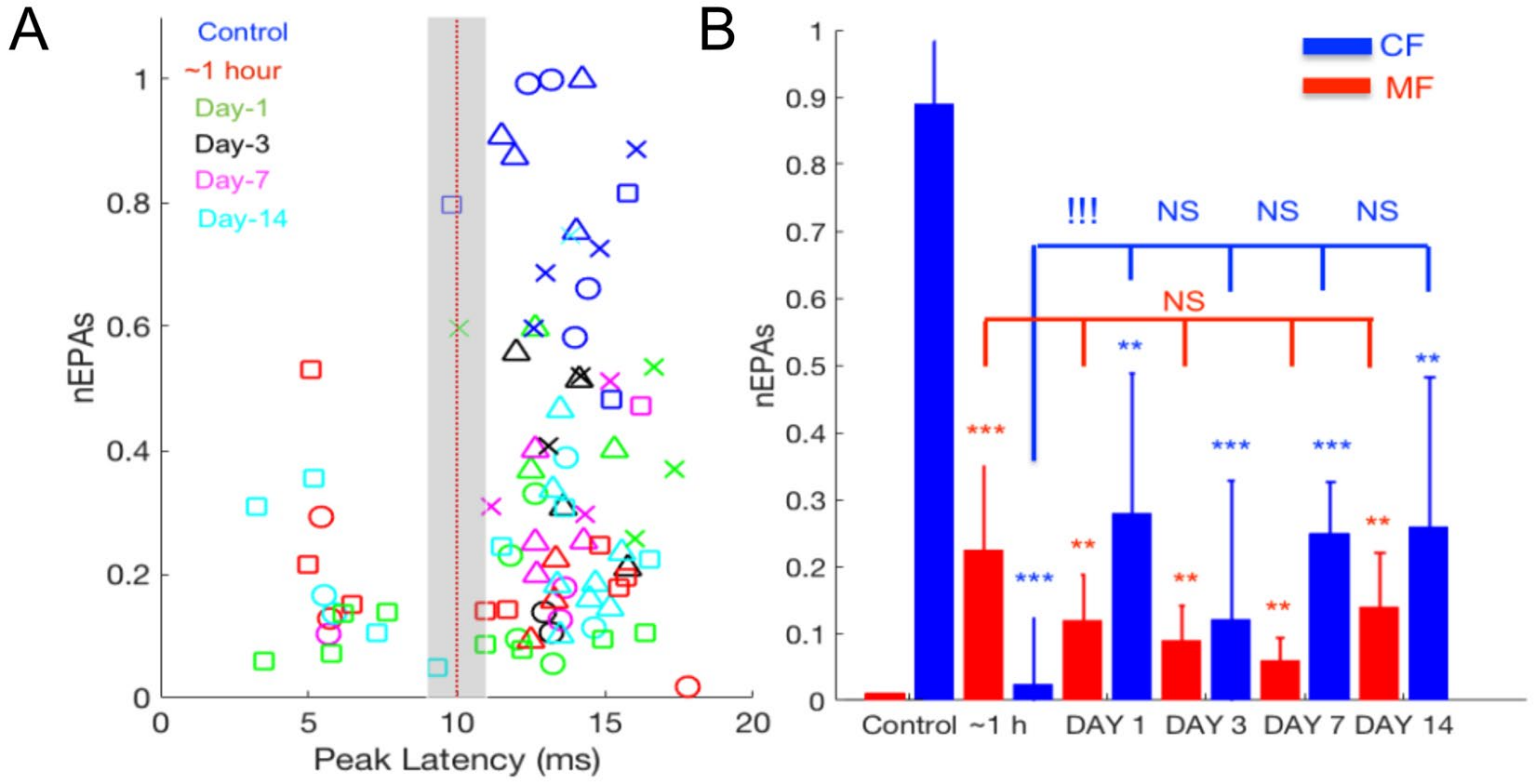
Excitability changes after injury in EP components by hand stimulation. (**A**) Amplitude distribution of MF (latency <10 ms) and CF (latency > 10 ms) components. EPAs were normalized (nEPA) to a maximum recorded amplitude on any day throughout the pre-/post injury period in each rat, and then a z-score value was applied as a threshold (Zscore > 2.05, 15 MFs and 71 CFs). The data were gathered from 4 rats as indicated by different marker shapes. While CF-EPAs indicated the strongest responses in the pre-injury (control) recordings in all animals (blue markers, T = 14/19 trials; Zscore > 2.05), MF-EPAs showed supra-threshold responses only in the post-injury period (T = 15/89 Trials, Zscore > 2.05), except day-3. (**B**) Same data with a lower threshold (Zscore > 1.55) were averaged and shown as mean ± s.d for MF (red) and CF (blue), respectively. Statistical analysis indicated significant increases in MF-nEPAs, and significant decreases in CF-nEPAs in the post-injury period (ANOVA followed by Dunnett’s test; control vs. post-injury time points, n = 5 days, n = 32 MF-nEPAs, n = 105 CF-nEPAs; ***P < 0.001, **P < 0.01, *P < 0.05). Individual days were compared using pairwise t-test (CF ~1 h vs. day-1; ^!!!^P = 0.0013, NS: Not significant).

As a measure of excitability in the cerebellar network, we used z-scores of the normalized amplitudes (nEPAs) of the MF- and CF-EPs (Fig. 5). In each animal, the majority of supra-threshold CF responses occurred before the injury (Fig. 5A; control, 90% or 19/21 trials in the pooled data). Conversely, none of the MF responses reached the excitability threshold on the day before the injury (Fig. 5A; Z_score_ > 2.05, no blue markers <10 ms, a gray area excluded). After the injury, the ratio of supra-threshold CF components declined to 66% (63/95 trials for all time points) from 90% (pre-injury), showing the sharpest decline within the hour (Fig. 5A; red markers). While all of the supra-threshold MF-nEPAs were registered in the post-injury period for all animals (Most heightened at ~1 hour; red markers, Z_score_ = 2.25 ± 0.11), CF-nEPAs were much stronger in the pre-injury period in comparison (blue markers in t ≥ 10 ms area, Z_score_ = 2.56 ± 0.13).

All supra-threshold responses (shown in Fig. 5A) were averaged for each day across animals and replotted (Fig. 5B). The z_score_ threshold was lowered to 1.55 for this particular analysis in order to include at least one data point per day. In each animal, the data set was normalized to the largest amplitude that was recorded during pre- or post-injury periods, following z-score qualification. Significant changes were detected in nEPAs of both MF and CF responses from pre- to post-injury (MF; F_(6,522)_ = 7.12, CF; F_(6,593)_ = 14.51) recordings. Within the first hour, CF-nEPAs were diminished almost completely down from 0.89 ± 0.09 in pre-injury. After day-1 of injury, the magnitudes of CF-nEPAs were still nearly 3-fold smaller (0.27 ± 0.2) than the pre-injury values and did not differ significantly on day-14 from day-7 (p = 0.22, one tailed t-test). Contrarily, MF-nEPAs increased to 0.22 ± 0.12 (p < 0.01; single data point from the pre-injury period in each rat) in the first hour and sustained higher magnitudes than the controls during the two weeks of the post-injury period (nEPAs = 0.14 ± 0.08 on day-14). These results suggest that the MF and CF amplitudes were also affected differentially by the injury, and analogous to the trend in their arrival times. Note that the overall effect of injury on the EP amplitudes was a net decrease by the AUC measures before separating the MF and CF components (Fig. 3).

### Ladder Walking

**Figure 6:**
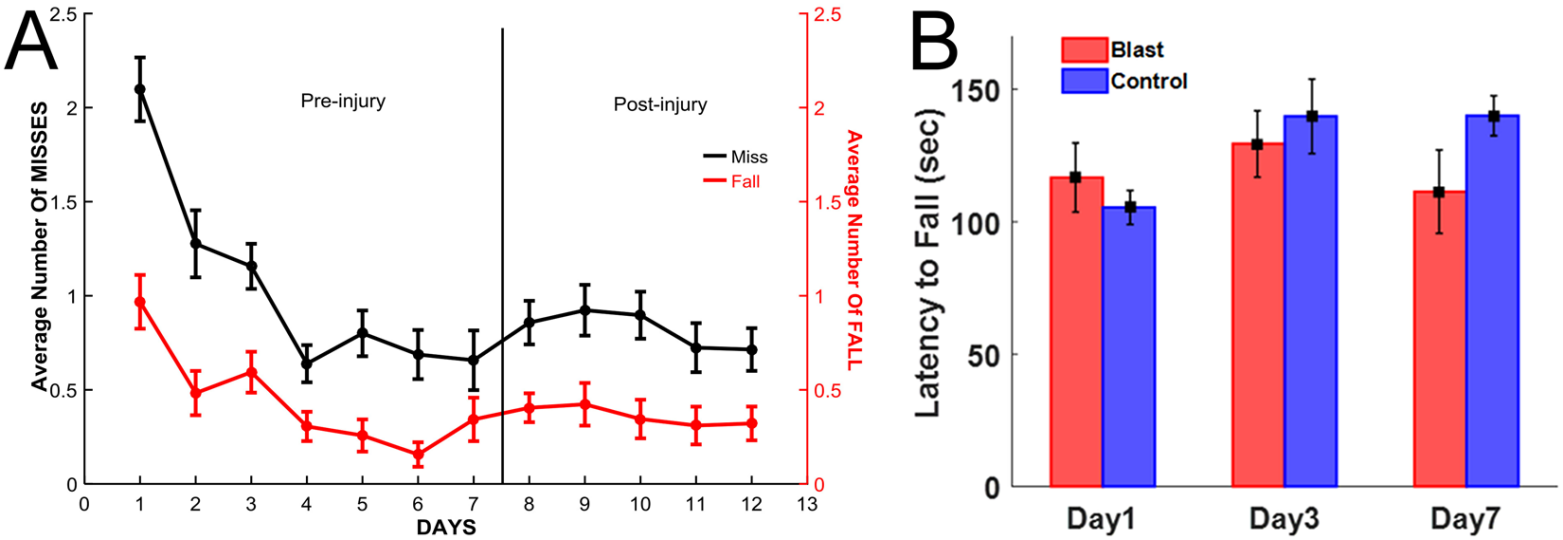
(**A**) Walking performances during repeated horizontal ladder crossings. Number of slips (misses) and falls in any of the four feet were scored in three rats. Rats demonstrated improved walking skills during the training period of one week with lowered number of falls and misses prior to injury (Days 1–7). Animals showed no statistically significant changes in motor performance scores with nearly the same miss and fall scores on day-1 (Day 8) and day-2 (Day 9) of injury. (**B**) Effect of mild blast injury on rotarod performance. Values were recorded from control and blast-injured animals on day 1, day 3 and day post-injury. Results are the mean ± SEM of the time animals remained on the rotarod before falling. There was no significant difference between control and injured animal groups at any time point (p > 0.05).

The functional impact of injury was assessed using the skilled locomotion test on the horizontal ladder rung (HLR) in a group of rats that were exposed to the same blast-pressures as the rats with array electrode implants (Fig. 6A, N = 3 rats). The walking performances improved during one-week of pre-injury training period as indicated by the lower number of foot slips (1.33 ± 0.28, p = 0.001; Tukey test, day-1 vs. day-4) and the body falls (1.44 ± 0.37, p = 0.009, Tukey test, day-1 vs. day-6). Interestingly, HLR scores did not present any significant differences after the blast injury (F_(2,32)_ = 0.909, p = 0.512; from day 4 to day 12). In fact, they showed nearly the same number of misses and falls on day-1 (Tukey test, p > 0.99; Day 7 vs Day 8) and day-2 (Tukey test, p > 0.95; Day 7 vs. Day 9) of the post-injury period. Results indicated no behavioral deficits in the limb functions during the learned HLR walking due to blast-exposure.

#### Rotarod tests

As another method to assess motor coordination in animals exposed to blast injury, rotarod testing was done in animals 24 h, 3 days and 7 days post-injury. Similar to ladder walking performance test, at any time point after the injury, latency times between control and injured animals did not display any statistically significant differences (Fig. 6B). These results are consistent with the ladder performance tests, and further confirms that electrophysiological measures are more sensitive tools to identify progressive cerebellar injuries following mild blast injury.

### Assessment of Neuronal Loss

**Figure 7:**
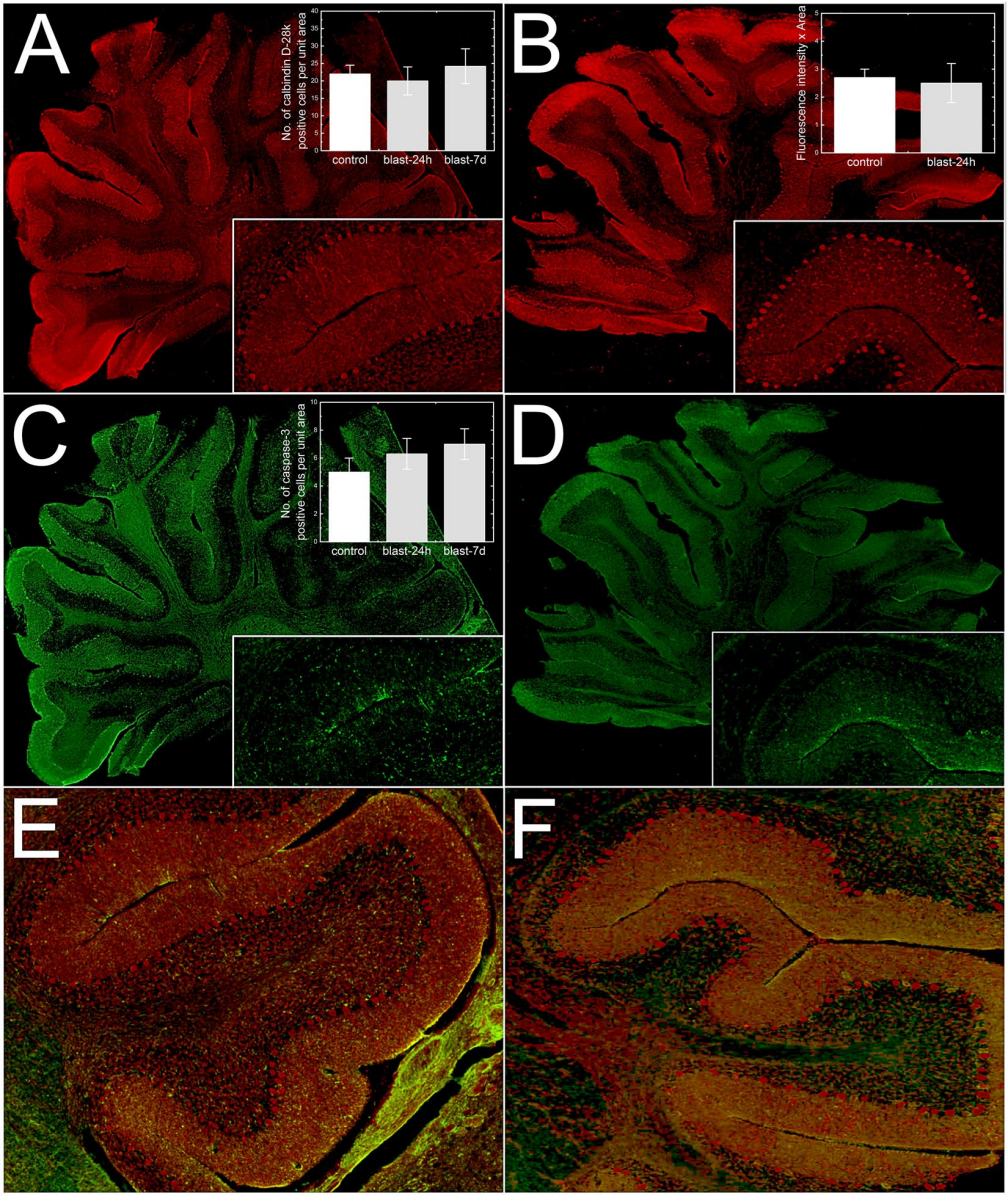
Immunofluorescence images of calbindin-D28K (**A**,**B**) and cleaved caspase-3 (**C**,**D**) in control, 24 hours and 7 days after blast injury. Sagittal sections with enlarged fragments from the paramedian lobules (insets) captured at 40x magnification and quantification results are presented. Paramedial lobules double stained for calbindin-D28K (red) and caspase-3 (green) showing no colocalization of caspase-3 in Purkinje cells (**E**,**F**). Bar plots present quantification of: 1) a cell count for: calbindin-28k (**A**), and capsase-3 (**C**) positive cells, and 2) calbindin-28k fluorescence signal (**B**). No appreciable changes in the levels of calbindin-D28K or cleaved caspase-3 were observed between control and the injured cerebella (p > 0.05).

In order to identify any potential neuronal loss contributing to electrophysiological disturbances at 24 h and 7 day post-injury, immunohistochemical analysis of calbindin D28K, an intracellular Ca^2+^ binding protein and a marker of Purkinje cells, as well as cleaved caspase-3, a marker of apoptosis was performed. Results indicate immunoreactivity (intensity) of calbindin D28K or a number of calbindin D-28K positive cells did not change in the paramedian lobule of cerebella in rats exposed to 130 kPa over pressure compared to controls (Fig. 7). Likewise, a number of caspase-3 positive cells did not change following injury. Calbindin D-28K and caspase-3 immunostaining were also performed 7 days post-injury and similar to 24 h data, no significant changes were observed between controls and 7-day post-injury animals. Additionally, to evaluate whether purkinje cells were undergoing apoptosis, we performed double immunostaining of calbindin-D28K and caspase-3. We did not observe any colocalization of caspase-3 positive staining in purkinje cells. These results indicate that Purkinje cell loss does not contribute to the observed disturbances in electrophysiological functions in this study.

## Discussion

### Electrophysiological Correlates of TBI

Electrophysiological detection of TBI related neurological abnormalities has been investigated in humans^22,23^ and animal models^24,25^. While researchers and clinicians mostly focused on the cerebral regions such as the neocortex^24,25^ and hippocampus^26–28^, reports on the cerebellum also presented strong evidence for the use of electrophysiology in TBI detection^20,29–31^. A wide spectrum of electrophysiological classifiers has been investigated. Nevertheless, amplitude and frequency analyses remain as the most sensitive parameters to identify abnormalities in the altered electrophysiology of the injured cerebellum. Amplitude variations in the electrical activity has previously been correlated to the cell loss in the cerebellum^20^, while the frequency^32^ and coherence spectra^33^ of the cerebellar oscillations have also been shown to change due to the injury.

Recently, we introduced the use of evoked potentials for detection of cerebellar injury in focal fluid percussion model with chronic implantation of multi-electrode arrays in rodents^30^. Our published data demonstrated the sensitivity of the electrophysiological assessment method in detection of cerebellar injuries earlier than the onset of molecular degeneration in fluid percussion injured (FPI) rats. Despite the differences in the injury mechanism between FPI-induced (both focal and locally diffuse) and blast-exposed (whole-brain diffuse) TBI models, cerebellar recordings showed a number of akin findings that support the sensitivity of the electrophysiological method. As a result of EPA amplitude variations between the animals, the recovery on day-3 (and day-7) was not statistically strong in the group data (Fig. 3) to unambiguously confirm the distinct recovery shown in the sample animal of Fig. 2. The amplitude analysis in conjunction with the time of arrivals (Fig. 5) suggests that the recovery is not complete within our observation window after injury. In contrast, we did not observe any signs of recovery in our previous FPI work within one week, which may be explained by different injury mechanisms.

Our definition of the MF time window was relaxed to account for additional propagation delays from periphery and we essentially used the first volley after the stimulus for the analysis. Here, we strictly defined the MF component with latencies less than 10 ms and extended our previous findings by separating the EP components into MF and CF mediated components. The former showed an increased and the latter showed a decreased excitability after the injury. Increased excitability in mossy fiber responses after FPI induction has been reported before. In Ai and Baker^20,29^, FPI applied over the posterior fossa immediately behind the lambda line in the center resulted in presynaptic hyperexcitability within mossy fiber granule cell synapses at 3 days post-injury along with by hyperexcitability of the parallel fiber-Purkinje cell synapses at 3–7 days post-injury. The major discrepancy between our and Ai and Baker’s results is that they did not observe any significant changes in neither of these synaptic strengths at 1 day after injury, a finding that was clearly suggested by our data with both FPI^30^ and blast injury mechanisms. A potential explanation was offered by our observation that the whisker EPs declined within the first hour post-injury as opposed to the hand EPs showing major changes on the next day. This suggests that different cerebellar networks may have varying time windows to respond to the injury depending on whether they are affected directly or indirectly.

The pre-implantation of an MEA may be a risk factor that the presence of the MEA on the cerebellar cortex during exposure to the blast wave may augment the impact and exacerbate the resulting injury. This concern was invalidated to a large extent by the fact that the whisker and hand evoked potentials were affected differentially and that the hand evoked signals did not differ significantly in recordings immediately after the injury. The deterioration of the signal amplitudes may still be occurring due to tissue encapsulation around the electrode until the chronic tissue response is complete in about two to three months^34–36^. The stability of the signals in a group of uninjured control animals with implantation times up to 21 days suggested that the signals do not deteriorate significantly within this time window^30^. The subdural implantation of the MEA can also provide a sub millimeter level spatial resolution for localization of injury if needed, as demonstrated by EP amplitude distributions from whisker and hand stimulation in uninjured animals^37^.

### Hand vs. Whisker Stimulation

Unlike the hand stimulation, the EPAs by whisker stimulation were significantly lower at each measurement point as seen in the pooled data from all animals (Fig. 3). This may be due to several factors. Cerebellar cortex contains multimodal functional topography^38–41^ that is defined by discrete clusters of neurons, which may receive multi-variant projections during stimulation of different body parts^38^. For instance afferent information from whisker activation is transmitted through a more direct and shorter pathway that involves trigeminal nuclei and inferior olive via middle cerebellar peduncle before reaching granule cells and PCs in the cerebellar cortex^42^; while inputs from the hand are mediated by spino-olivary pathways ascending via inferior cerebellar peduncle to activate the same type of neurons in the cerebellum^43–45^. Biomechanical factors during blast injury might induce differential effects on the hand and whisker networks of the cerebellum that are defined by different ascending pathways. This is supported by studies indicating a higher sensitivity of the vestibular nucleus to shock-wave induced blast injuries in humans and rats^46–48^, although we cannot confirm a similar effect since we did not include behavioral tests to verify balance and coordination deficits in our experimental animals. Nevertheless, it is possible that an adjacent structure to the vestibular fibers, the trigeminal tract, may also have a higher sensitivity to the blast-waves.

Additionally, we observed large differences in EPAs, particularly in those from the hand, between the animals that were exposed to blast pressures with lower (100 psi) and higher (130 psi) pressure peaks (data not shown). While the whisker EPAs showed rapid drops within hours of injury, only at higher pressures the hand EPAs presented discernible deteriorations. While the differences in the measured peak pressures explain the variations in our pooled data, this observation also suggests the sensitivity of the cerebellar EPs to graded levels of injury severity. In our blast experiments the animals were held in prone position with shock waves traveling from rostral to caudal direction and reaching the cerebellum last. In FPI experiments, the injury is usually induced just above the cerebellum. Though the blast loading is biomechanically distinct from FPI, the subtle electrophysiological changes were measurable in both injury types.

### MF vs. CF Mediated Components

Cerebellar evoked potentials were previously investigated with penetrating as well as surface electrodes and characteristic waves (amplitude, onset time, duration, etc.) were linked to cerebellar morphology^37,49–52^. The sole output of the cerebellar cortex, the Purkinje cells (PCs), receive inputs through two major pathways. First, the mossy fibers (MF) that project on PCs via granule cell (GC)-parallel fiber (Pf) pathway and generate an early (2–6 ms; onset latency, P1-N1, N2) excitatory post synaptic potentials (EPSPs) in the evoked potential waveform. Second, the climbing fibers (CF) that originate from the inferior olive (IO) and make strong excitatory connections with the PCs and give rise to local field deflections with 8–20 ms latencies in response to a sensory stimulus^52,53^.

The evoked potentials, identified by their anatomical origins within the cerebellar cortex, were shown to detect changes of excitability^54,55^. We separated the cerebellar EPs into mossy and climbing fiber components with respect to their arrival times (Figs 4 and 5). While the CF components presented delayed arrival times (up to ~4 ms) for all days of post-injury, the MF responses had a changing trend. We noted that the MF onsets were initially increased (~1 h), then decreased (day 1) and finally increased again from day-3 to day-14 of the injury period. Finally, the increasing standard deviation of the CF delays between the recording channels indicated a wider spatial distribution of arrival times across the cerebellar cortex.

Differential effects in the timing of the MF and CF signals prompted a similar analysis to be performed on the amplitudes. Typically, the MF deflections exhibited smaller amplitudes with negative polarity, which is plausible considering the weakness of PF-PC synapses. The effect of the CF pathway on PC dendrites is much stronger with hundreds of synapses^56^, and thus the CF component is recorded with larger amplitudes and positive polarity. Figure 5 suggests an increase in the MF mediated volleys and a decrease in the CF mediated components during the post-injury period, particularly at early stages, from 1 hour to a day. The higher z-score threshold yielded many more MF component detections after the injury suggests hyperexcitability. Increased excitability of pre-synaptic inputs at the mossy fibers-granule cell terminals was reported before^20^. Although our findings suggested a similar trend, we argue that the time window for MF hyperexcitability may be occurring in the earlier acute stage (hours to a day) rather than in the delayed phase (day-3 to day-7) of injury.

### Behavioral Tests

We hypothesized that electrophysiological approach could be sensitive enough to investigate mild injuries that would be too subtle to detect with conventional methods. To confirm this hypothesis, we first evaluated the behavioral scores during a skilled walking task on a horizontal ladder rung. The cerebellum plays a major role in motor learning tasks when the predictive timing and coordination is essential^57,58^. Animal models suggest that cerebellar injury can lead to Purkinje cell losses and behavioral deficits in moderate to severe head injuries^17,59^. We implemented a horizontal ladder rung test to evaluate skilled walking parameters such as foot slips, step cycle, limb coordination to complement foot fault scorings in pre- and post-injury periods. Although multiple parameters were analyzed (not shown), we observed only clear improvements in the reduction of foot slips and partial (or complete) falls in the learning period prior to injury induction. After blast exposure, animals showed no differences in their walking performance on the ladder. In our previous study, FPI at 15–30 psi induced detectable changes in step duration, suggesting that the HLR task is a proper behavioral paradigm to demonstrate cerebellar injuries^60^. The absence of walking alterations in the HLR task among blast injury cohort suggests that the cerebellum is affected only mildly. These results show that the electrophysiological method can detect changes in the cerebellar circuits even in the absence of behavioral deficits.

### Immunohistochemistry

Neuronal loss, particularly the Purkinje cell loss has been reported in different forms of TBI with different injury severities^18^. Earlier, employing fluid percussion model of TBI, we also observed that mild injury (~15 psi) caused a significant Purkinje cell loss (decreased calbindin D28K immunoreactivity and increased Fluoro-Jade C staining) in parasagittal sections of cerebellar cortex 7 days post-injury^30^. Our previous observation is in contrast with our present study showing no Purkinje cell loss following blast injury at 24 h or 7 days post-injury, although the peak over pressure appear to be similar (15 psi = 103 kPa). It is possible that the injury outcomes may be dictated by the mode of injury (e.g., blast vs. blunt injury). Accordingly, direct tissue injury at the epicenter in the blunt TBI could trigger a cascade of events (neuroinflammation, local blood brain barrier breakdown) that may be different from blast injury. Additionally, a single blast at moderate levels may not be sufficient to trigger neurodegeneration whereas a single blunt impact and associated direct tissue damage could render Purkinje neurons more vulnerable. In fact, employing a repetitive blast model in mice (3 blasts at 19 psi over 24 h interval) chronic Purkinje cell loss and persistent synaptic disturbances occurred^12^, which supports our tenet. Nevertheless, electrophysiological disturbances observed in the present study appear to be not due to neuronal loss.

## Methods

### Implant Surgery

Custom-design flexible multi-electrode arrays (E32_250_25_30_PML, NeuroNexus, MI) were implanted using sterile surgical techniques in 10 Long Evans rats (male, 250–275 g) one week prior to blast exposure. Data from 7 rats that were collected at matching time points in the post-injury period are presented here. All procedures were approved and performed in accordance to the guidelines of the Institutional Animal Care and Use Committee, Rutgers University, Newark, NJ. Anesthesia was induced using isoflurane (4% in 100% O_2_) and maintained with lower doses of the same (1.5–2.5%). Dexamethasone sodium phosphate was administered (2 mg/kg, IM) before surgery to reduce cerebellar edema. A craniotomy of ~2 mm^2^ was made over the right cerebellum (corner at 1.7 mm lateral from midline and 2.5 mm caudal from the posterior edge of the skull) in order to implant the flexible MEA subdurally on the paramedian lobule (PML). The platinum contacts (30 µm diam.) of the MEA had a pitch of 200 µm and 250 µm in AP and ML directions respectively in 4 × 10 configuration (Fig. 1). The MEA substrate was a thin polymer (polyimide, thickness 12 µm) and fabricated in a slightly curved shape to fit between the blood vessels without occluding them and cover most of the PML. Contact impedances varied between 800 kΩ–1.2 MΩ in saline prior to implantation and stabilized around 1–1.5 MΩ after the first week of surgery. A large reference electrode was incorporated into the MEA at one corner to suppress common-mode signals originating from subcortical locations within the cerebellum. The leftmost edge of the array was juxtaposed to the paravermal vein on the right PML. Trace amounts of octyl cyanoacrylate tissue adhesive (Nexaband, WPI, Inc., FL) was applied on the edges of the MEA to fix it on the pia surface before covering it with autologous connective tissue for sealing the dural opening. The micro connector at the end of the MEA ribbon cable was fixed to the skull using octyl cyanoacrylate first as an adhesive layer over the skull followed by dental acrylic.

### Blast-Wave Injury

Seven to ten days were allowed for the animals to recover from the surgery and the evoked potentials to stabilize before the injury. Then, the rats were exposed to a single blast wave in a 6 m long shock tube with 9-inch square cross-section located in the Center of Injury Biomechanics, Materials and Medicine, NJIT^61^. The shock tube generates blast overpressure vs. time pulses that have been validated against live-fire explosions so that the experimental conditions reported here are both field-validated and realistic^62^. Rats were anesthetized with a cocktail of ketamine and xylazine (100 mg/10 mg/kg, IP) before mounting them inside the test chamber located 2.80 m from the point where the shock-wave was generated at one end of the tube and 3.05 m from its exit. The incident blast pressures, as measured near the test subject, varied between 110 and 130 kPa, had a duration of 5.7 ± 0.3 ms, and corresponding impulse values, i.e. the area under the overpressure curve, of 234 ± 27 Pa·s. As in previously reported experiments^63,64^, animals were exposed to the blast in a horizontal head-on position while strapped to an aerodynamic aluminum holder with a thin cotton cloth wrapped around the body. By this experimental setting the gross head motion was eliminated almost entirely as confirmed by high-speed video recording. The high-speed videos were recorded on Photron FASTCAM Mini UX100 camera equipped with Tokina 100 mm f/2.8 macro lens and operating at a frame rate of 5000 fps. Typically, 8000 frames of video footage were recorded in a single experiment and subsequently saved via Photron FASTCAM Viewer 3.3.5 software. The incident overpressure was recorded at the location of the specimen in the test chamber using a LabView program running on a customized data acquisition system based on National Instruments PXI-6133 S-Series DAQ Module. The pressure waveform was recorded using PCB Piezotronics sensors model 134A24 (Depew, NY) at 1.0 MHz sampling frequency for a duration of 100 ms.

### Electrophysiological Signals

This study utilized the sensory evoked potentials as a measure of electrophysiological intactness of the cerebellar cortex in a rat model. Sensory evoked potentials were elicited by a mechanical stimulation device, a 1 mm diameter cylindrical wood stick attached to the center of an audio speaker and activated by a short-pulse through a computer with milli-second accuracy. The control pulse was passed through a high-voltage solenoid driver circuit (SDM840, Magnetic Sensor Systems, CA) to achieve a fast and large displacement (3–4 mm) of the speaker coil. A train of mechanical stimuli (20 stimuli at 1 pps) were delivered to ipsilateral whiskers (not necessary the same one in each session) and the back of the hand to evoke local field potentials in the PML cortex of the cerebellum also under ketamine/xylazine anesthesia (30 mg/kg and 2 mg/kg, IP). The neural recordings were performed in a large Faraday cage through a 34-channel headstage amplifier (Gain 800, 0.8 Hz–3 kHz, Triangular Biosystems, NC). Signals were sampled at 16 kHz and filtered at 10 Hz-500 Hz in Matlab (Mathworks). Stimulus-triggered averaging (STA) was employed to suppress the background noise. Further details of electrophysiological methods were previously described elsewhere^30,37^.

### Characterization of EPs

Evoked potentials were characterized by amplitude (EPA) and latencies following the time of stimulus arrival, marked as 0 ms in Fig. 2A. Two different methods were followed for EPA quantification. First, we calculated the area under the curve (AUC) of any potential deflections that is above five times the standard deviation of the baseline activity in the post-stimulus time window, i.e. 0–50 ms (shaded areas of top right panel in Fig. 3A). Any signal component arriving 50 ms after the stimulus was discarded since these may be the responses to the stimuli relayed through the brain centers outside the cerebellar network, such as those from the cerebral cortex^65,66^.

In the second method, evoked volleys were first identified as either mossy fiber (MF) or climbing fiber (CF) mediated potentials based on the timing of their peaks (before or after 10 ms, left Figs 4–5). Then, it was determined whether the MF and CF volleys were significantly greater (Z_score_ > 2.05) than the baseline fluctuation. Those peak values that exceeded three times the background standard deviation was taken. If there was more than one volley for each type, only the largest deflection was included in the analysis. All quantitative measures used the EPAs normalized (nEPA) by the maximum EPA ever recorded before or after injury in that given animal.

### Behavioral Testing

Three additional rats of the same size, strain, and gender as in the MEA implants were used for the behavioral testing. Walking on horizontal ladder rung (HLR) was chosen as the behavioral paradigm in order to investigate the impact of blast injury on the motor function. The HLR method is sensitive to the injury of the cerebellum^67^. The custom-designed ladder was assembled by 90 × 19 cm Plexiglas side walls that were separated by metal rungs of 3 mm diameter. The spacing between the rungs was set to 1 cm (regular) for habituation and 1–3 cm (irregular) in the training sessions. The ladder was elevated to 30 cm from the ground and two video cameras (220 × 330 pixels, 100 fps, Allied Vision) were installed to image the entire terrain while being able to see the hands and the feet without obstruction. Both ends of the ladder had a chamber where the animal could receive a food reward. During the first week, animals became accustomed to the environment and slowly learned to cross the ladder. Each rat had two ~10 min sessions per day for 7 days, at which point all animals learned to cross the ladder without hesitation. After this pre-training, the ladder rung pattern was changed by removing some of the rungs randomly and their walking was scored by counting the steps in which they missed the rung momentarily but corrected quickly (miss) or lost their balance before placing the hand correctly (fall). The same random rung pattern was used for all rats. Then, the animals were injured using a single blast wave at ~130 kPa, as in the other group of rats for electrophysiological recordings. Behavioral data collection sessions consisted of 10 crossings on each day of pre- and post-injury periods. The video recordings were analyzed frame-by-frame using VirtualDub software. The number of missed steps and falls were counted for each crossing and used for statistical analysis in IBM SPSS Statistics software.

### Rotarod tests

We also performed rotarod performance test as an additional measure to investigate the effect of blast injury on motor function. A set of 12 rats were subjected to pretraining for 3 days twice daily (with 30 min resting period) to acclimate to the testing procedures before exposing the animals to blast injury. During the testing phase, rotarod was set in the accelerated mode with increasing number of revolutions from 4–40 rpm reaching within 90 s (2.5 s intervals for 1 rpm increment). The average time each rat spent on the rotarod during the 3-day pertaining was calculated and the values were statistically validated using nonparametric test to meet non-gaussian distribution to eliminate bias in the distribution of animals (with approximately equal latency times in control and experimental groups) before exposing the animals to blast injury. Rats were then exposed to blast injury and rotarod testing was performed at 24 hours, 3- and 7-days post-injury and results were expressed as mean ± SEM of latency time.

### Immunohistochemistry

Three rats Long Evans rats (male, 250–275 g) were subjected to a single blast injury with 130 kPa peak overpressure and animals were euthanized 24 hours post-TBI for the immunological analysis. Three sham control rats of the same size, strain and gender received anesthesia and noise exposure but without blast exposure, i.e. anesthetized animals were placed next to the shock tube on the outside and then a single shot was fired. Following blast injury, animals were monitored closely for any signs of trauma-related distress such as apnea.

### Immunofluorescence and microscopy

Twenty-four-hour and 7 days post-injury, both sham and TBI animals (4 animals in each group for 24 h study and 6 animals in each group for 7-day study) were transcardially perfused with PBS followed by 4% paraformaldehyde. Cerebella were dissected and post-fixed in 4% paraformaldehyde (PFA) for additional 48 h and cryoprotected by immersing in 30% sucrose. Fixed cerebellum was vertically cut into two halves and left half of the cerebellum from control or blast animals was embedded in OCT (Optimal Cutting Temperature) in sagittal orientation and quickly frozen in isopentane cooled to liquid nitrogen temperature. 15 μm thick slices were collected 3 mm from the surface of the embedded frozen cerebellum blocks, using Leica CM3050 cryostat and mounted on glass slides. At least 3 sections from each animal cut serially were mounted on each slide. Sections were washed with 10 mM phosphate buffered saline (PBS), fixed in ice-cold methanol (100%) solution for 10 min at −20 °C. The tissue sections were blocked with 10% donkey serum at room temperature for 1 hour in PBS containing 0.03% Triton X-100. Fixed tissues were incubated with anti-calbindin D-28K (rabbit polyclonal, Calbiochem, 1:300) or anti-cleaved caspase-3 (rabbit polyclonal, Millipore Inc, 1:100) overnight at 4 °C, followed by incubation (1 h) with Alexa Fluor 594 secondary antibody. We also performed a double immunostaining of calbindin D-28K (mouse monoclonal, Calbiochem, 1:400) and caspase-3 (Rabbit polyclonal, Millipore 1:100). The tissue was counterstained with DAPI (Invitrogen, Carlsbad, CA) to visualize cellular nuclei and facilitate quantification (cell count).

### Image acquisition and analysis

Slides were digitized (40x magnification) using a Leica Aperio Versa 200 fluorescent microscope. For fluorescence intensity quantification, exposure times and grey scale balance were adjusted manually for each channel prior to scanning. Fluorescence intensity of calbindin D-28K cerebellar sections (2–3 in each slide) derived from 3 individual animals/group was quantitated using a FLAreaQuantV1 algorithm (Leica Biosystems) and expressed as average fluorescence intensity per unit area. Briefly, in each image, a minimum intensity threshold value was set that will exclude any background fluorescence caused by nonspecific binding of the fluorescent secondary antibody. This was followed by setting a maximum intensity threshold as to remove any oversaturation due to excess fluorescent dye. The area quantification algorithm then determines if the intensity value of each pixel within the specified region falls between the minimum and maximum intensity thresholds. The algorithm outputs the area of positive stain for each brain region, the average intensity of each channel, and expression profile of the protein. For visualization of calbindin D-28K and caspase-3 positive cells, ImageJ software (NIH) was used. Briefly, color images were converted to gray scale and different regions of interest (ROIs, 10–15 random areas of approximately 4 mm^2^ diameter) were drawn in the paramedian lobe, and the images were thresholded to separate background from particles of interest, in this instance, number of calbindin D-28K and caspase-3 positive particles which different ROIs with varying area were counted, averaged and represented as total number of cells/unit area.

## Conclusions

The acute phases of injury were monitored *in vivo* using local field potentials recorded with chronically implanted micro-electrode arrays. The results demonstrate the sensitivity of the electrophysiological measures for detection of cerebellar injuries at levels of blast pressure that are not detectable by behavioral (ladder walking) or immunohistological assays. The two main findings are: First, the largest modulations of the EP amplitudes were seen within the first 24 hours after injury, which was followed by a slower pace of recovery period. Second, blast-wave injury produced differential effects both on the amplitudes and arrival times of the MF and CF mediated components. Taken together, we can conclude that micro EcoG method can identify subtle neurological changes triggered by mTBI conditions.
